# Angiogenesis Inhibition by a Short 13 Amino Acid Peptide Sequence of Tetrastatin, the α4(IV) NC1 Domain of Collagen IV

**DOI:** 10.3389/fcell.2020.00775

**Published:** 2020-08-11

**Authors:** Alexia Vautrin-Glabik, Jérôme Devy, Camille Bour, Stéphanie Baud, Laurence Choulier, Anthony Hoarau, Aurélie Dupont-Deshorgue, Christèle Sellier, Bertrand Brassart, Jean-Baptiste Oudart, Laurent Ramont, Jean Claude Monboisse, Sylvie Brassart-Pasco

**Affiliations:** ^1^Laboratoire de Biochimie, Université de Reims Champagne-Ardenne (URCA), Reims, France; ^2^CNRS UMR 7369, Matrice Extracellulaire et Dynamique Cellulaire (MEDyC), Reims, France; ^3^Plateau de Modélisation Moléculaire Multi-échelle, URCA, Reims, France; ^4^CNRS UMR 7021, Laboratoire de Bioimagerie et Pathologies, Université de Strasbourg, Illkirch, France; ^5^CHU Reims, Service Biochimie-Pharmacologie-Toxicologie, Reims, France

**Keywords:** angiogenesis, matrikine, Tetrastatin, integrin, alpha 5 beta 1 collagen IV

## Abstract

Angiogenesis is defined as the formation of new capillaries by sprouting from the pre-existing microvasculature. It occurs in physiological and pathological processes particularly in tumor growth and metastasis. α1, α2, α3, and α6 NC1 domains from type IV collagen were reported to inhibit tumor angiogenesis. We previously demonstrated that the α4 NC1 domain from type IV collagen, named Tetrastatin, inhibited tumor growth in a mouse melanoma model. The inhibitory activity was located in a 13 amino acid sequence named QS-13. In the present paper, we demonstrate that QS-13 decreases VEGF-induced-angiogenesis *in vivo* using the Matrigel plug model. Fluorescence molecular tomography allows the measurement of a 65% decrease in Matrigel plug angiogenesis following QS-13 administration. The results are confirmed by CD31 microvessel density analysis on Matrigel plug slices. QS-13 peptide decreases Human Umbilical Vein Endothelial Cells (HUVEC) migration and pseudotube formation *in vitro*. Relevant QS-13 conformations were obtained from molecular dynamics simulations and docking. A putative interaction of QS-13 with α_5_β_1_ integrin was investigated. The interaction was confirmed by affinity chromatography, solid phase assay, and surface plasmon resonance. QS-13 binding site on α_5_β_1_ integrin is located in close vicinity to the RGD binding site, as demonstrated by competition assays. Collectively, our results suggest that QS-13 exhibits a mighty anti-angiogenic activity that could be used in cancer treatment and other pathologies with excessive angiogenesis such as hemangioma, psoriasis or diabetes.

## Introduction

Angiogenesis is the formation of new blood vessels and capillaries from sprouting of pre-existing blood vessels. It normally occurs during wound healing and embryonic development, but it is also required for tumor growth and metastasis. This process is regulated by growth factors, such as vascular endothelial growth factors (VEGFs), which bind to their receptors on the normal endothelial cell surface, induce transduction pathways and promote proliferation and migration of endothelial cells and vascular tube formation (Teleanu et al., 2019).

In recent years, the basement membrane (BM), a specialized extracellular matrix (ECM), has been recognized as a key regulator of cell behavior, and not only as an architectural support. BM is an important structural and functional component of blood vessels (Silva et al., 2018). Several BM components were reported to largely participate in the regulation of tumor angiogenesis (Kalluri, 2003).

Basement membrane are composed of type IV collagen in association with other minor collagens, such as collagens XVIII or XIX, laminins, nidogens, and perlecan. Type IV collagen is composed of three α(IV) chains, out of six possible [α1(IV)-α6(IV)]. Each α(IV) chain comprises a 7S N-terminal domain, a long interrupted triple helical domain and a globular C-terminal non-collagenous (NC1) domain (Wu and Ge, 2019). The α1(IV) NC1 domain (Arresten), the α2(IV) NC1 domain (Canstatin), the α3(IV) NC1 domain (Tumstatin), and the a6(IV) NC1 domain were reported to inhibit angiogenesis (Petitclerc et al., 2000; Wietecha et al., 2013; Monboisse et al., 2014). The α4(IV) NC1 domain was reported to slightly decrease bFGF-induced angiogenesis while α5(IV) NC1 domain has no effect (Petitclerc et al., 2000). Karagiannis and Popel reported the inhibitory effects of Pentastatin-1 and -2, two α5(IV) NC1 domain-derived peptides, on Human Umbilical Vein Endothelial Cells (HUVEC) proliferation (Karagiannis and Popel, 2008). They also pointed out the inhibitory effects of Tetrastatin-2 and Pentastatin-3, two peptides from the α4(IV) and α5(IV) NC1 domains, respectively, on VEGF-induced HUVEC migration We previously demonstrated the potent anti-tumor activity of the α4(IV) NC1 domain, named Tetrastatin (Brassart-Pasco et al., 2012). We recently identified the minimal active sequence (QKISRCQVCVKYS: QS-13) of Tetrastatin that reproduced whole Tetrastatin anti-tumor properties *in vivo* and *in vitro* on melanoma cell proliferation, migration, and invasion (Lambert et al., 2018).

In the present article, we investigated the inhibitory effects of QS-13 on angiogenesis *in vivo* in a Matrigel plug assay and *in vitro* on HUVEC proliferation, migration and pseudotube formation.

## Materials and Methods

### Peptide Synthesis

QS-13 was purchased from Proteogenix^®^ (Schiltigheim, France). It was obtained by solid-phase synthesis using a FMOC [N-(9-fluorenyl) methoxy-carbonyl] derivative procedure. It was then purified by reverse phase high performance liquid chromatography using a C18 column, eluted by a gradient of acetonitrile in trifluoroacetic acid and lyophilized. Its purity (>98%) was assessed by HPLC and mass spectroscopy.

### Matrigel Plug Angiogenesis Assay

Eight-week-old albinos female B6(C)Rj -Tyr c/c mice were purchased from Janvier Laboratories (Saint-Berthevin, France). The animals were fed *ad libitum* with a chlorophyll-free diet 10 days before and during imaging experiments. The study was performed in compliance with “The French Animal Welfare Act” and following “The French Board for Animal Experiments.” Experiments were conducted under approval of the French “Ministère de l’Enseignement Supérieur et de la Recherche” (Ethics Committees Nos. C2EA-56 and C2EA-75) in compliance with the “Directive 2010/63/UE”. Protocol no. 4373_V1 APAFIS (07/09/2016).

Four hundred μL of Matrigel mix composed of growth factor-reduced Matrigel (Dutscher, Brumath, France) supplemented with 100 ng/mL recombinant mouse VEGF (R&D System-Bio-Techne, Lille, France), 350 ng/mL of recombinant mouse bFGF (R&D System-Bio-Techne, Lille, France) and 25 UI/mL of Heparin (R&D System-Bio-Techne, Lille, France) were injected into the left flank of each mouse. Mice were divided into two groups of 8 mice: positive control (Matrigel mix), QS-13-treated mice (Matrigel mix + 40 μM QS-13). Positive control mice received PBS and QS-13 treated mice received QS-13 (10 mg/kg) intraperitoneally at days 3, 7, and 11.

### *In vivo* Fluorescence Imaging

At day 13, 100 μL of AngioSense680^TM^ (PerkinElmer, Inc., United States) were injected into the right orbital plexus of mice. At day 14, mice were anesthetized with 2% isoflurane and images were obtained with a fluorescence molecular tomographic (FMT^®^) imaging system (FMT4000, PerkinElmer, Inc., United States). Then, mice were sacrificed. Matrigel plug were removed and placed into 4% formaldehyde for histological analyses. 3D reconstruction and image analysis were performed using TrueQuant^TM^ software (PerkinElmer, Inc., United States).

### CD31 Immunostaining

CD31 immunostaining were performed on 4 μm thick Matrigel plug sections. After deparaffinization, sections were incubated with a Tris–EDTA buffer, pH 8.4 for 20 min at 97°C, washed with distilled water and then incubated with hydrogen peroxide blocking solution (Abcam, Paris, France) for 10 min at room temperature and washed with PBS. They were incubated with Protein Block (Abcam) for 10 min at room temperature, washed with PBS, and incubated overnight at 4°C with an anti-CD31 rabbit monoclonal antibody diluted 1/1000 (ab 28364 from Abcam) and washed again with PBS. The first antibody was detected using the Rabbit specific HRP/DAB (ABC) Detection IHC Kit (Abcam) according to the manufacturer’s instructions. Sections were counterstained with hematoxylin (Novocastra). To evaluate MicroVessel Density (MVD), three sections per plug were performed at three different depth levels of the plug and three different fields were acquired under an inverted microscope. Each positive endothelial cell cluster of immunoreactivity in contact with the selected field was counted as an individual vessel in addition to the morphologically identifiable vessels with a lumen, according to Weidner’s method (Weidner, 1995).

### Cell Culture

Human Umbilical Vein Endothelial Cells were purchased from Promocell (Heidelberg, Germany). Cells were grown in Endothelial Cell Growth Medium (ECGM, Promocell, Heidelberg, Germany) at 37°C in a humid atmosphere with 5% CO_2_ in air. At 70–90% confluency, cells were subcultured according to Promocell subcultivation protocol, using the Detach Kit (Hepes BSS, Trypsin/EDTA, Trypsin Neutralization Solution). They were used before passage 5.

### Proliferation Assay

For cell proliferation measurement, 2,000 HUVECs were seeded in 96-well plates and cultivated in ECGM supplemented with 10 ng/mL of Vascular Endothelial Growth Factor (VEGF-165, Promocell) with or without 40 μM QS-13. After 24, 48, 72, and 96 h, cell proliferation was measured using the WST-1 cell proliferation reagent (Sigma, Saint-Quentin-Fallavier, France), according to the manufacturer’s instructions. Absorbance was read at 450 nm using a Biochrom Asys UVM 340 microplate reader (Biochrom, Yvelines, France).

### Scratch Wound Assay

Human Umbilical Vein Endothelial Cells were seeded in 24-well plates and cultivated to confluence in ECGM at 37°C in a humid atmosphere (5% CO_2_, 95% air). At confluence, a homogenous wound was created in each well with a sterile 1,000 μL pipet tip. After washing, cells were incubated with fresh ECGM supplemented with 10 ng/mL VEGF with or without 40 μM QS-13 for 24 h. The wound area was measured at the beginning (T0) and end of the experiment (T24h) using the ImageJ analysis program (NIH, Bethesda, MD, United States) and the percentage of wound closure after 24 h was calculated.

### Pseudotube Formation on Matrigel

The ability of HUVECs to form capillary tube structures was evaluated on Matrigel (Dutscher, Brumath, France). Matrigel (200 μL/well of a 10 mg/mL solution) was allowed to polymerize at 37°C for 30 min. After 30 min, 50,000 cells were suspended in ECGM supplemented with 10 ng/mL of VEGF with or without 40 μM QS-13 and seeded into each well. Plates were incubated at 37°C in a humid atmosphere (5% CO_2_, 95% air) for 6 h. Capillary tube formation was imaged after 6 h under an inverted microscope. Quantitative evaluation of the capillary tubes was performed with ImageJ software using the Angiogenesis Analyzer tool. The number of master junctions, master segments, meshes, and the total mesh area of the capillary tube structures were determined.

### Adhesion Assays

Cells were detached with 50 mM Hepes, 125 mM NaCl, 5 mM KCl, and 1 mM EDTA, washed three times with ECGM, pre-incubated for 30 min with effectors [mouse anti-human α_5_β_1_, catalog number 555614 from BD Pharmingen (10 μg/mL), irrelevant IgG (10 μg/mL) or RGDS peptide 20 mg/mL (Sigma)]. 10,000 cells were seeded per well of a 96 well-plate previously coated with QS-13 and saturated with 1% BSA. After 60 min, cells were washed three times with HEPES buffered Balanced Salt Solution, fixed with 1.1% glutaraldehyde solution and stained with 1% crystal violet solution. Staining was extracted with 10% acetic acid and absorbance was read at 560 nm.

### Affinity Chromatography

Human Umbilical Vein Endothelial Cells were cultured in 150 cm^2^ culture flasks until 70% confluence and cell layer was scrapped in RIPA buffer (Sigma, St Quentin Fallavier, France) supplemented with a protease inhibitor cocktail (Halt Protease Inhibitor Cocktail, Thermo Fisher Scientific, Illkirch, France). Cell lysate was incubated for 30 min at 4°C and centrifuged at 10,000 *g* for 10 min at 4°C to remove insoluble debris. Concentration of soluble proteins was quantified using Biorad Protein Assay (BioRad, Marnes-La-Coquette, France) according to the manufacturer’s instructions. Chromatography was performed on a HiTrap NHS-activated Sepharose High Performance column (GE Healthcare, Orsay, France) functionalized with QS-13 according to the manufacturer’s instructions. Protein extract was chromatographed at 4°C. Unbound proteins were removed with 30 mL of washing buffer [10 mM Tris, 1 mM CaCl_2_, 1 mM MgCl_2_, pH 7.6 supplemented with PIC (ProteoBlock Protease Inhibitor Cocktail, Fermentas, Illkirch, France; w/v) and 0.1% (w/v) octylglucoside]. Proteins bound to the affinity column were eluted with a buffer containing 10 mM Tris, pH 7.6, 0.1% (w/v) octylglucoside and PIC, supplemented with increasing concentrations of NaCl (0.15, 0.6, and 1 M). SDS sample buffer containing 10 mM DTT was added to eluted proteins; samples were incubated for 30 min at 37°C, denatured for 5 min at 95°C and electrophoresed in a 0.1% SDS, 10% polyacrylamide gel. They were then transferred onto Immobilon-P membranes (Millipore, St Quentin en Yvelines, France). Membranes were blocked with 5% non-fat dry milk, 0.1% Tween 20 in TBS for 2 h at room temperature, incubated overnight at 4°C with a rabbit anti-β_1_ integrin polyclonal antibody (AB1952P, Merck Millipore) or a rabbit anti-α_5_ integrin polyclonal antibody (#4705, Cell Signaling) diluted 1/1000 in 1% non-fat dry milk, 0.1% Tween 20 in TBS) and then for 1 h at room temperature with a second peroxidase-conjugated anti-IgG antibody. Immune complexes were visualized with the ECL chemiluminescence detection kit (GE Healthcare, Orsay, France).

### Solid Phase Assay for Studying QS-13/Integrin Interaction

Wells of a 96-well plate were coated with 25 nM α_5_β_1_ integrin (3230-A5-050; R&D Systems, Lille, France) in amounts overnight at room temperature. The coating was then blocked with TBS containing 5% dry milk, 1 mM MgCl_2_, and 1 mM CaCl_2_ for 2 h at room temperature. After washing three times with washing buffer (0.1% dry milk, 1 mM MgCl_2_, and 1 mM CaCl_2_ in TBS), the plate was incubated for 90 min at room temperature with 100 μL per well of ranging from 1.25 to 20.10^–11^ moles/well biotinylated-QS-13 diluted in washing buffer. After 3 washes, 100 μL of streptavidin-peroxidase diluted 1/20000 in washing buffer were added to each well and incubated for 15 min at room temperature. After 4 washes, 100 μL per well of tetramethylbenzidine (TMB), a peroxidase substrate, were added and incubated in the dark for 15 min. The enzymatic reaction was stopped by adding 50 μL per well of 0.5 M H_2_SO_4_. The intensity of the yellow coloration was measured at 450 nm with a Biochrom Asys UVM 340 microplate reader.

For competition experiments, wells of a 96-well plate were coated with 25 nM α_5_β_1_ integrin and then blocked as described above. Wells were then incubated for 90 min at room temperature with biotinylated QS-13 with or without unbiotinylated QS-13 (molar ratio ranging from 1/1 to 250/1) diluted in washing buffer. The following steps are as described above.

### Surface Plasmon Resonance Analysis

All experiments were performed on a Biacore T200 instrument (GE Healthcare) at 25°C. Sensor surfaces and other Biacore consumables were purchased from GE Healthcare. Integrin α_5_β_1_ was from R&D Systems. The running buffer, HEPES buffered saline (HBS, composed of 0.01 M HEPES, pH 7.4, 0.15 M NaCl, 3.4 mM EDTA) was filtered through a 0.22 μm membrane and supplemented with 0.05% P20. Biotinylated peptides were captured on streptavidin-coated sensor chips. Briefly, CM5 sensor chips (GE Healthcare) were preconditioned by duplicate injections of 10 mM HCl, 50 mM NaOH, each for 10 s, and water for 20 s. Before covalent immobilization of streptavidin, traces of biotinylated products that could remain in the flow system were neutralized by injecting a streptavidin solution (0.1 mg/mL in running buffer) for 5 min through all flow cells (Baltzinger et al., 2013). Streptavidin was then stably immobilized using standard amine-coupling methods. The flow rate was 10 μL/min. Surfaces were activated by injection of a 1:1 mix of 0.2 M N-ethyl-N’-(3-dimethylaminopropyl)-carbodiimide hydrochloride (EDC) and 0.05 M N-hydroxysuccinimide (NHS) for 10 min, followed by a 5 min injection of streptavidin at 200 μg/mL in 10 mM sodium acetate (pH 5) and then deactivated with ethanolamine-HCl (pH 8.5) for 10 min. The surface was then subjected to four pulses (100 μL) of 50 mM NaOH at a flow rate 50 μL/min to wash out all non-covalently bound streptavidin. Biotinylated QS-13 (QKISRCQVCVKYSK-biot) was injected onto streptavidin at 5 μg/mL in running buffer, for 10 s at a flow rate of 100 μL/min. Responses were stabilized by five pulse injections of 50 mM NaOH at a flow rate 50 μL/min. Reference surfaces (Fc1) were treated similarly except that an irrelevant biotinylated peptide was injected. Eleven different concentrations of α_5_β_1_ integrin (0.12, 0.25, 0.5, 1, 2, 4, 8, 16, 32, 64, and 130 nM) were injected into the flow cells at 30 μL/min for 300 s. Dissociation was followed for 600 s. Binding curves were double-reference substracted from buffer blank and reference flow cell (Fc 1). The equilibrium response (Req) was recorded 5 s before the end of integrin injection. The K_D_ was determined by fitting the equilibrium response versus the [integrin] curve to a simple 1:1 interaction model with the Biacore T200 evaluation software (GE Healthcare).

### Docking Experiments

Docking of QS-13 onto α_5_β_1_ integrin (RCSB Protein Data Bank 3VI3) was performed using Autodock software (version 4.2; Morris et al., 2009). The docking parameters were as previously described (Lambert et al., 2018). The software was used with a fixed integrin and semi-flexible QS-13 ligand (the backbone was frozen as well as the amide links and guanidinium groups). Because the integrin is a large molecule, we performed several independent dockings targeting different subvolumes of the protein; we considered 125 overlapping boxes with a volume of 47.25 Å × 47.25 Å × 47.25 Å. Each box was divided along the three directions, and the distance between the nodes was equal to 0.375 Å. The Lamarckian genetic algorithm was used, and for each ligand, 150 dockings were performed with the default parameters of Autodock except for the population size (150), number of energy evaluations (5 × 10^6^), and maximum number of generations (30,000; Lambert et al., 2018). Molecular models were derived from the preliminary study. Molecular models were graphed with VMD software, which is available online.

### Statistical Analyses

For *in vitro* experiments, results are expressed as the mean ± SD and statistical significance were determined using Student’s *t*-test. For *in vivo* experiments, the non-parametric Mann–Whitney test was performed.

## Results

### QS-13 Decreases *in vivo* Matrigel Plug Angiogenesis

**FIGURE 1:**
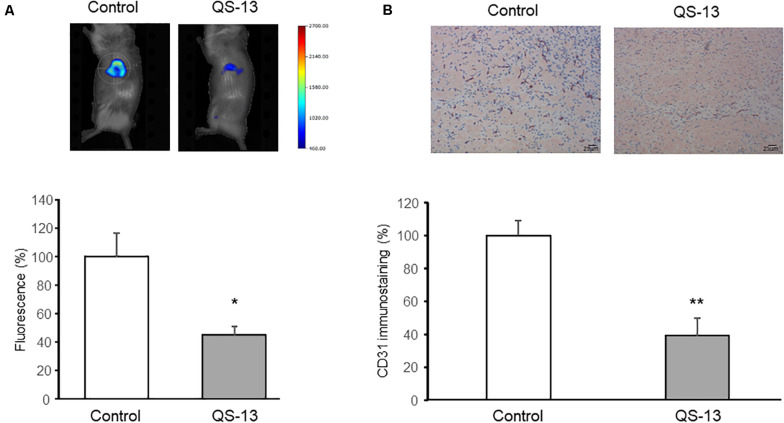
QS-13 decreases *in vivo* angiogenesis in a Matrigel plug model. Matrigel (400 μL) was subcutaneously injected into mice. After 13 days of treatment with VEGF alone or with VEGF and QS-13, a fluorescent imaging agent was administrated. **(A)** At day 14, anesthetized mice were imaged using molecular fluorescence tomography and FMT signal intensities were measured. **(B)** Matrigel plugs were then excised, CD31-immunostaining was performed on Matrigel plug sections and microvessel density (MVD) was evaluated. Two independent experiments (*N* = 2) were carried out with 4 mice in each group (*n* = 4). The histograms represent the means of the two experiments ± SD. *p < 0.05; **p < 0.01.

The subcutaneous Matrigel plug assay in mouse is a gold standard *in vivo* assay to screen pro- or anti-angiogenic molecules. Administration of a fluorescent imaging agent (AngioSense680^TM^) and quantitative analyses with a fluorescence molecular tomographic (FMT) imaging system revealed a significant decrease (−57%) of angiogenesis in Matrigel plug of QS-13-treated mice compared to control mice at day 13 (Figure 1A). The Matrigel plugs were then excised and 4 μm thick sections were performed and stained using an anti-CD31 antibody as endothelial cell marker. The MVD analysis of the different sections confirmed that QS-13 inhibits *in vivo* angiogenesis by 61% (Figure 1B).

### QS-13 Decreases *in vitro* Endothelial Cell Migration and Pseudotube Formation Without Affecting Cell Proliferation

In order to decipher the anti-angiogenic effect of QS-13, we performed *in vitro* studies.

**FIGURE 2:**
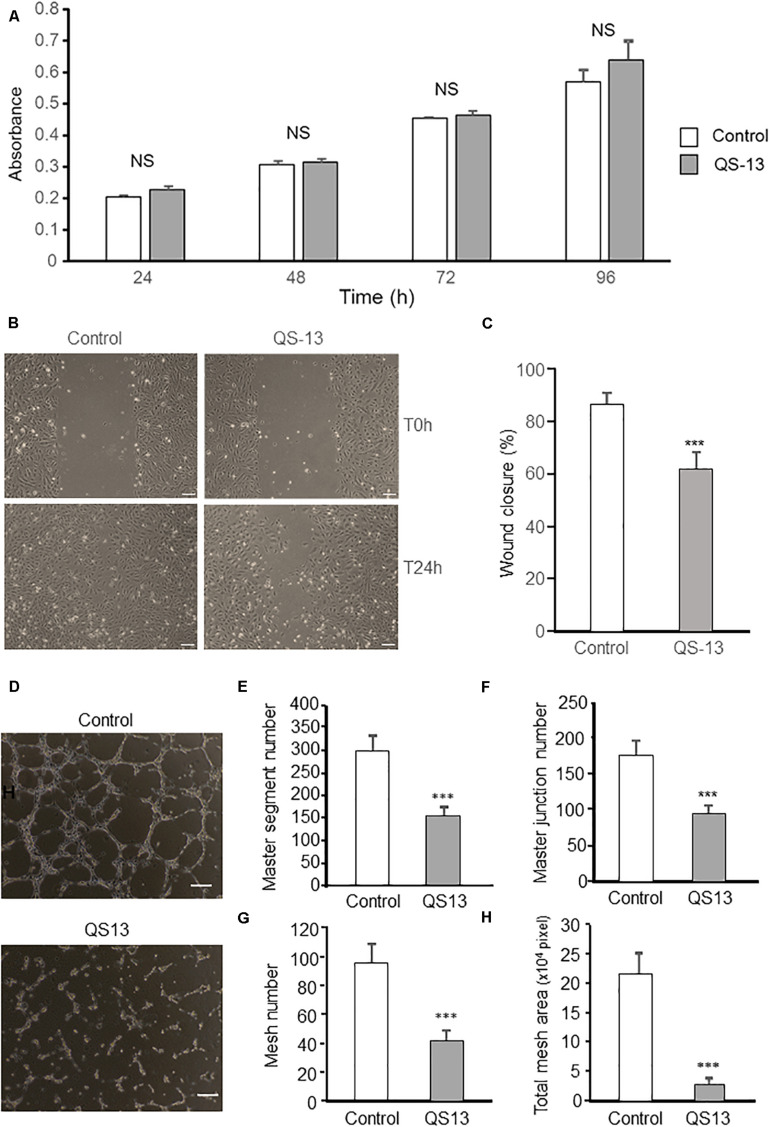
QS-13 decreases *in vitro* endothelial cell migration and pseudotube formation without affecting cell proliferation. To study cell proliferation, HUVECs were incubated for 24, 48, 72, and 96 h with or without 40 μM QS-13. **(A)** Proliferation was measured using the WST-1 reagent and absorbance was read at 450 nm (*n* = 8). Three independent experiments were carried out. The histogram represents the means ± SD of the more representative one. NS, not significant. To study endothelial cell migration, a scratch wound was performed using a pipet tip at confluence. HUVECs were then incubated with or without 40 μM QS-13 for 24 h at 37°C. **(B)** Wounds were microphotographed after 24 h of incubation. Scale bar: 50 nm. **(C)** Wound closure was measured using ImageJ software. Three independent experiments were carried out. The histogram represents the means ± SD of the more representative one. *n* = 8, ***p < 0.001. To study pseudotube formation, HUVECs were seeded on Matrigel coated well and incubated with or without 40 μM QS-13. **(D)** Pseudotube formation was observed under an inverted microscope after 6 h and photographed. Scale bar: 200 nm. **(E)** The number of master segments, **(F)** the number of master junction, **(G)** the number of meshes and **(H)** the total mesh area of pseudotube were determined with ImageJ software. Three independent experiments were carried out. The histogram represents the means ± SD of the more representative one. *n* = 8, ***p < 0.001.

As proliferation of endothelial cells plays an essential role in angiogenesis, HUVEC proliferation under the influence of QS13 was assessed at 24, 48, 72, and 96 h using the WST-1 method. QS-13 does not significantly alter HUVEC proliferation up to 96 h incubation (Figure 2A).

Human Umbilical Vein Endothelial Cell migration, a key step in angiogenesis, was studied using the scratch wound healing model. Scratch wounds were photographed at 0 and 24 h (Figure 2B). Compared to control, QS-13 decreased wound closure by 28% (Figure 2C).

Human Umbilical Vein Endothelial Cell pseudotube-formation assay is a well-established *in vitro* angiogenesis assay based on the ability of endothelial cells to form three-dimensional capillary-like tubular structures. We demonstrated that QS-13 strongly altered pseudotube formation (Figure 2D): it decreased the number of master segments by 47% (Figure 2E), the number of master junction by 44% (Figure 2F), the number of meshes by 56% (Figure 2G) and the total mesh area by 87% (Figure 2H).

### Identification of α_5_β_1_ Integrin as a QS-13 Receptor on HUVECs

#### *In silico* Studies

**FIGURE 3:**
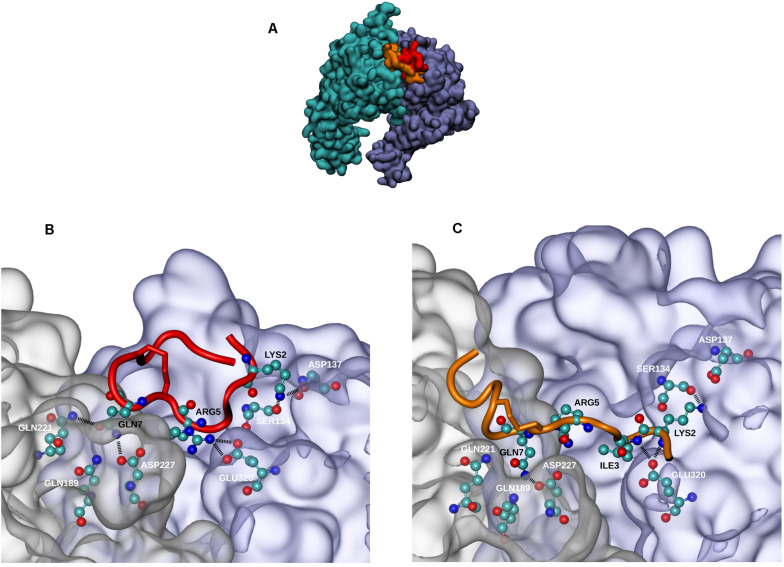
Docking experiments of QS-13 on α_5_β_1_ integrin. The best results of QS-13 docking experiment display the same interaction area with the α_5_β_1_ integrin at the interface between the two integrin subunits. **(A)** Integrin subunits and QS-13 are represented using the Quick Surface scheme and colored according to the nature of the chain (cyan for α_5_ and magenta for β_1_) or the conformation of QS-13 (first conformation extracted from the MD in red and second conformation extracted from the MD in orange). **(B,C)** Zoomed-in representations of the best energy conformation 1 and 2 of QS-13, respectively. The New Cartoon scheme is used to represent QS-13 and the Quick Surface scheme used for integrin is set to transparent. Residues making contacts between integrin and QS-13 (a contact is defined as a distance between two atoms lower than 3 Å) are represented with the CPK mode. Color labels are adopted in order to distinguish integrin (white) and QS-13 (black) residues. Hydrogen bonds are highlighted with thick black dashed lines.

α_5_β_1_ integrin was previously reported to be largely expressed in HUVECs. The integrin mediates cell adhesion to ECM (Short et al., 1998; Zeng et al., 2009) and cell migration through a VEGFR-dependent mechanism (Orecchia et al., 2003). Thereby, we investigated a putative interaction of QS-13 with the α_5_β_1_ integrin. We previously performed molecular dynamics simulations on isolated QS-13 (Lambert et al., 2018) and observed that a disulfide bond locked the CQVC sequence in a conformation that exposed the glutamine (Q) side chain. The presence of the disulfide bond was confirmed by MALDI-ToF MS analyses. Molecular docking experiments were carried out to test the hypothesis of a QS-13/α_5_β_1_ integrin interaction (Figure 3). For the two considered conformations of QS-13 peptide (extracted from molecular dynamics simulations), the best 180 results (from the energy point of view) were used in order to cluster the solutions and to identify the main interaction areas. For each conformation, the most populated cluster (32.2% and 19.4% for conformation 1 and 2, respectively) contained the pose associated to the best free energy of binding (-5.71 kcal/mol and -7.25 kcal/mol for conformation 1 and 2, respectively). The visualization of these poses (Figure 3A) demonstrated that both conformations share the same interaction area with the α_5_β_1_ integrin at the interface between the two integrin subunits. Despite the fact that the two QS-13 shapes were rather different [one could be considered “folded” (Figure 3B) and the second “elongated” (Figure 3C)], the contacts they made with the integrin were very similar. Indeed, the key interactions established by QS-13 with the α_5_β_1_ integrin mainly involved the following residues: the first lysine (K2) with S134 and D137 of the β_1_ integrin subunit; the arginine (R5) with D227 of the α_5_ integrin subunit and E320 of the β_1_ integrin subunit; the glutamine (Q7) exposed by the disulfide bond with Q189, Q221, and D227 of the β_1_ integrin subunit. The number of inter-molecule hydrogen bonds is comparable from one pose to the other, and two pairs of interaction are conserved, namely S134/K2 and D227/Q7.

#### *In vitro* Experiments

**FIGURE 4:**
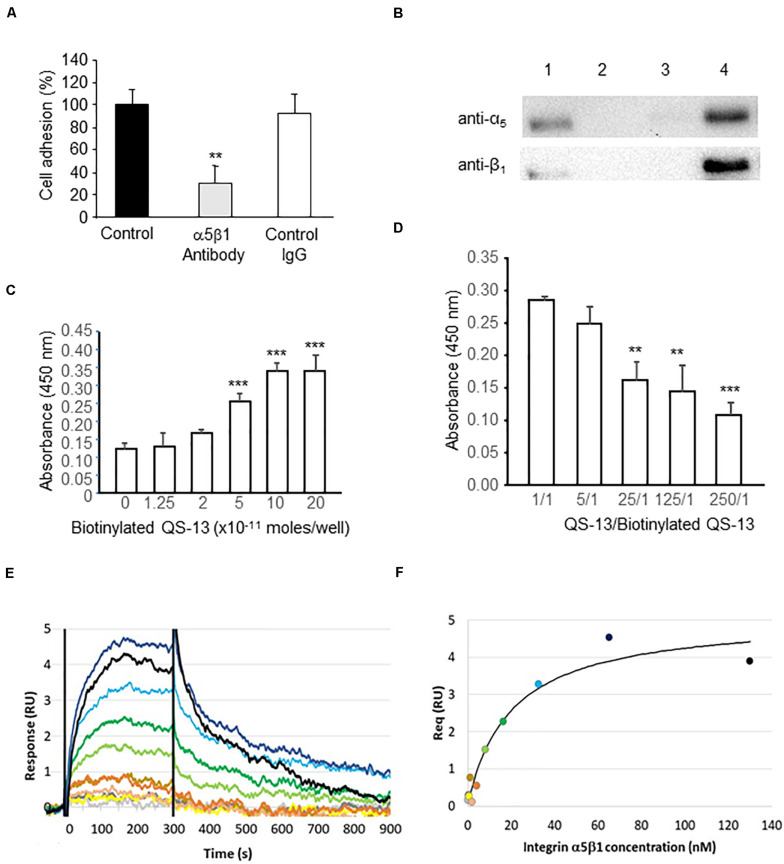
QS-13 peptide binds to α_5_β_1_ integrin. **(A)** HUVECs were pre-incubated with ECGM alone, ECGM containing an anti-α_5_β_1_ blocking antibody or an irrelevant antibody (10 μg/mL). HUVEC adhesion was measured as described in the section “Materials and Methods.” Three independent experiments were carried out. The histogram represents the means ± SD of the more representative one (*n* = 8), **p < 0.01. **(B)** HUVEC extracts were submitted to affinity chromatography on a QS-13 -bound column. Lane 1: total cell extracts; lane 2: unbound proteins; lane 3: 0.15 M NaCl eluted fraction; lane 4: 0.6 M NaCl eluted fraction. **(C)** Direct interaction between α_5_β_1_ integrin at concentrations ranging from 0.25 to 2.10^– 10^ mole/well and QS-13 was studied using solid phase assay as described in the “Materials and Methods” section. Two independent experiments were carried out (*n* = 4), ***p < 0.001. **(D)** A competition assay was performed using increasing concentrations of unbiotinylated-QS-13 while biotinylated QS-13 and α_5_β_1_ integrin concentrations were kept constant Two independent experiments were carried out (*n* = 4), **p < 0.01, ***p < 0.001. **(E,F)** The equilibrium affinity of the α_5_β_1_ integrin-QS-13 interaction was analyzed by SPR. **(E)** Concentration-dependent responses of integrin α_5_β_1_ (0.12 to 130 nM range) to the streptavidin-captured biotinylated QS-13. **(F)** Fit of the equilibrium response (Req) versus [α_5_β_1_ integrin] to a 1:1 binding indicates a K_D_ of 20.3 ± 7.5 nM.

To determine whether QS-13 binds to HUVECs through α_5_β_1_ integrin, we measured HUVEC adhesion on QS-13 in the presence or absence of an anti-α_5_β_1_ integrin blocking antibody (10 μg/mL). The preincubation of HUVECs with the blocking anti-α_5_β_1_ antibody significantly inhibited (71%) cell adhesion on QS-13, whereas an irrelevant IgG had no effect (Figure 4A), suggesting a putative interaction between QS-13 and α_5_β_1_ integrin. To verify this hypothesis, we performed affinity chromatography. HUVEC extracts were loaded onto a QS-13-functionalized affinity column. Proteins bound to the affinity column were eluted with increasing concentrations of NaCl (0.15, 0.6, and 1.0 M; Supplementary Figure S1). Eluted samples, analyzed by western blot, showed the presence of α_5_ and β_1_ integrin subunits in the 0.6 M eluted fraction (Figure 4B). The existence of a direct interaction between α_5_β_1_ integrin and QS-13 was investigated using two different methods: solid phase assay and surface plasmon resonance (SPR). For this purpose, QS-13 was biotinylated at its C-terminus. In the solid phase assay, we demonstrated that biotinylated-QS-13 binds to α_5_β_1_ integrin in a ligand concentration dependent manner (Figure 4C). Biotinylated QS-13 was covalently immobilized on a streptavidin-coated CM5 sensor chip. α_5_β_1_ integrin was then injected at eleven concentrations ranging from 0.12 to 130 nM. Moreover, a competitive assay was performed to confirm the specificity of the interaction. Increasing amounts of unbiotinylated QS-13 decreased biotinylated-QS-13 binding to α_5_β_1_ integrin (Figure 4D). In SPR experiments, biotinylated-QS-13 was covalently immobilized on a streptavidin-coated CM5 sensor chip. α_5_β_1_ integrin was then injected at eleven concentrations ranging from 0.12 to 130 nM. Figure 4E presents the sensorgrams obtained after double referencing (subtraction of reference channel and buffer injection). α_5_β_1_ integrin binds to Q-S13 in a dose-dependent manner. The double referenced equilibrium responses recorded 5 s before the end of integrin injections were called Req. Figure 4F shows the plotted responses of Req as a function of α_5_β_1_ integrin concentration. The equilibrium affinity parameter (K_D_) was determined by fitting the Req versus the [α_5_β_1_ integrin] curve to a simple 1:1 interaction model. We measured a K_D_ of 20.3 ± 7.5 nM for the QS-13/α_5_β_1_ integrin interaction.

Taken together, the results confirm that QS-13 directly binds to α_5_β_1_ integrin.

### RGDS Peptide Competes With QS-13 for α_5_β_1_ Integrin Binding on HUVECs

**FIGURE 5:**
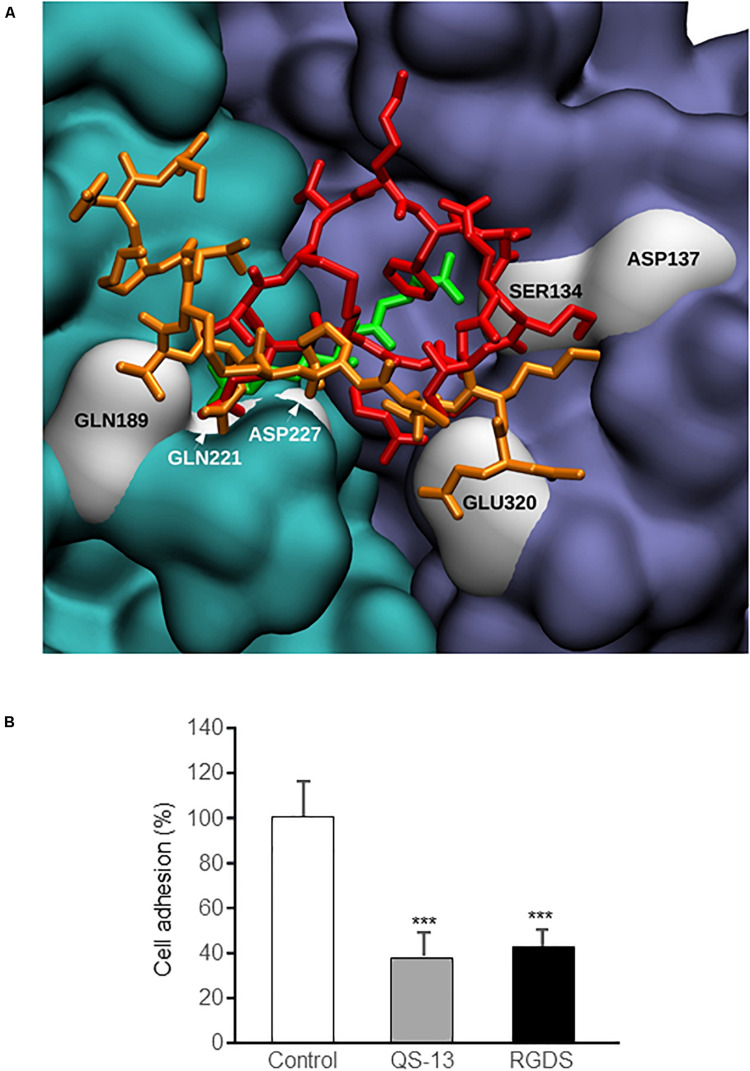
Comparison of QS-13 and RGD theoretical binding sites on α_5_β_1_ integrin and *in vitro* confirmation. **(A)** α_5_β_1_ integrin is represented using the Quick Surface scheme and a color code related to the nature of the subunit (cyan for α_5_ and magenta for β_1_). The best docking poses of the two QS-13 conformations are superimposed with the conformation of the RDG peptide (from the experimental complex corresponding to the PDB ID 3VI4 solved with X-ray experiments) and depicted with a color coded (first QS-13 conformation extracted from the MD in red, the second in orange and RGD peptide in green) Licorice representation. Residues from the integrin making contacts with QS-13 (as evidenced in Figure 3) are highlighted with white surface representation and labeled. **(B)** HUVECs were pre-incubated for 30 min with culture medium alone (control), culture medium supplemented with RGDS peptide (20 μg/mL) or with QS-13 (20 μg/mL) and adhesion was measured. Three independent experiments were carried out. The histogram represents the means ± SD of the more representative one. *n* = 8, ***p < 0.001.

As specified in the “Materials and Methods” section, docking experiments were carried out using the structure associated with PDB ID 3VI3: this crystallographic data corresponded to the ligand-free form of α_5_β_1_ integrin. The structure associated with PBD ID 3VI4 was related to α_5_β_1_ integrin headpiece in complex with RGD peptide: the visualization and comparison of most likely QS-13 conformations with this experimental ligand-associated form (Figure 5A and Supplementary Figure S2) evidenced that the QS-13 binding site was in close vicinity to the RGD peptide binding site. In the case of the RGD peptide, the residues implicated in the interactions were the arginine (R) with S134 of the β_1_ integrin sub-unit and the aspartic acid (D) with Q189, Q221, and D227 of the α_5_ integrin sub-unit. Even though the sizes of the RGD peptide and of QS-13 were very different, the same residues are involved in both interactions with the integrin subunits.

To test this hypothesis, competition binding assay were performed. HUVECs were pre-incubated with medium alone or medium supplemented with either QS-13 or RGDS peptide and seeded on QS-13 coating. Cell pre-incubation with RGDS inhibited cell adhesion the same way as QS-13 itself (-60%) (Figure 5B).

## Discussion

Angiogenesis plays critical roles in human physiological processes. This is a complex phenomenon regulated in a spatial and temporal manner that depends on the cooperation between angiogenic factors, ECM components, and endothelial cells. Uncontrolled angiogenesis may lead to several angiogenic disorders and to vascular overgrowth (hemangiomas, psoriasis, vascularized tumors…). Numerous pro-angiogenic drivers have been identified such as VEGF. To date, the most common approaches to the inhibition of the VEGF axis include the blockade of VEGF receptors (VEGFRs) or ligands by neutralizing antibodies, as well as the inhibition of receptor tyrosine kinases (RTK). Unfortunately, this type of inhibitors leads to numerous side effects as well as resistance phenomena (Haibe et al., 2020).

In the present article, we demonstrate that the Tetrastatin QKISRCQVCVKYS peptide sequence (QS-13) inhibits *in vivo* angiogenesis in the Matrigel plug model. *In vitro*, QS-13 does not affect cell proliferation but decreases cell migration and pseudotube organization on Matrigel. The preincubation of HUVECs with a blocking anti-α_5_β_1_ antibody significantly inhibited cell adhesion on QS-13, whereas an irrelevant IgG had no effect, suggesting an interaction between QS-13 and the α_5_β_1_ integrin.

The NC1 domains of the different α(IV) collagen chains were reported to exert anti-tumor or anti-angiogenic effects (Mundel and Kalluri, 2007; Monboisse et al., 2014; Brassart-Pasco et al., 2020). We and others demonstrated that the anti-tumor activities of Tumstatin or Tetrastatin were mediated through binding to α_v_β_3_ integrin at the tumor cell surface (Pasco et al., 2000; Brassart-Pasco et al., 2012; Lambert et al., 2018). It was also demonstrated that Arresten, Canstatin or Tumstatin also exerted anti-angiogenic activities mediated through binding to α_1_β_1_, α_1_β_1_/α_v_β_3_/α_v_β_5_, and α_v_β_5_/α_v_β_3_ integrin binding, respectively (Brassart-Pasco et al., 2020). Furthermore, several integrins, including α_5_β_1_, and α_v_β_3_/β_5_, have been described to play an important role in tumor angiogenesis. Their overexpression on tumor neo-vessels suggest new anti-angiogenic therapies (Schaffner et al., 2013).

The anti-α_5_β_1_ integrin antibody M200/volociximab was reported to inhibit angiogenesis and to suppress tumor growth and metastasis in mice (Almokadem and Belani, 2012). It showed preliminary evidence of efficacy in advanced NSCLC (Besse et al., 2013).

Peptides also emerged as important therapeutic agents in angiogenesis-dependent diseases due to their low toxicity and high specificity (Rosca et al., 2011). The α_5_β_1_-blocking peptide ATN-161, derived from the synergy region of fibronectin, showed preclinical anti-cancer activities (Stoeltzing et al., 2003; Khalili et al., 2006). It entered clinical testing but failed to provide therapeutic benefits (Cianfrocca et al., 2006).

Another matrikine, a 20-kDa C-terminal fragment of type XVIII collagen, endostatin, was also reported to inhibit α_5_β_1_ integrin (Sudhakar et al., 2003). It was tested in clinical studies in combination with chemotherapies and radiotherapies but the results were inconsistent, probably due to recombinant protein production (Alday-Parejo et al., 2019).

We also demonstrated that the inhibitory effects of Tetrastatin are conformation-dependent with a crucial role of the presence of a disulfide bond in QS-13 (Lambert et al., 2018). Because of the influence and importance of QS-13 disulfide bond, the present *in silico* investigation of the interaction between α_5_β_1_ integrin and QS-13 was designed using peptide conformations displaying the presence of the disulfide bond. The results of the docking experiments demonstrate that QS-13 is able to bind to α_5_β_1_ integrin in a stable mode since the evaluated free energy of binding of the best solutions is below the threshold of -3.00 kcal/mol. In addition to the energy aspect, the statistical analysis of the best molecular docking results indicates that the interaction areas with α_5_β_1_ integrin are not randomly distributed. Indeed, the clustering of the best poses leads to the identification of a region gathering the highest number of results and corresponds to an interface region between the two integrin subunits. This region overlaps with the binding site of the RGD sequence, a well-known integrin recognition sequence. Despite the size difference between RGD and QS-13, the residues involved in the interaction at the protein level show a strong overlap and are either polar residues or residues with charged side chains prone to hydrogen-bonding. It should also be noted that in the case of QS-13, the glutamine residue exposed by the presence of the disulfide bond (Lambert et al., 2018), is one of the three residues in contact with the integrin.

The interaction between QS-13 and α_5_β_1_ integrin is confirmed by affinity chromatography, solid phase binding assay and SPR. As the RGD binding site was shown to overlap QS-13 theoretical binding sites determined by molecular docking, a competition experiment was performed *in vitro*. Cell pre-incubation with RGDS inhibited cell adhesion to the QS-13 by about 60%, confirming that QS-13 binding site was in close vicinity to RGD binding site on α_5_β_1_ integrin.

Taken together, our results demonstrate that QS-13 binds to α_5_β_1_ integrin and inhibits endothelial cell migration and angiogenesis and is a potent anti-angiogenic agent. Since the disulfide bond forms spontaneously in solution, QS-13 may be protected from protease degradation *in vivo*. It offers new therapeutic strategies in hemangiomas and psoriasis treatment alone or in combination with anti-microbial peptides (Morizane and Gallo, 2012).

In tumors, α_5_β_1_ integrin is overexpressed and represents an interesting target for the administration of anti-cancer agents *in situ* (Schaffner et al., 2013; Blandin et al., 2015; Alday-Parejo et al., 2019). In addition to its inhibitory effects on endothelial cell migration and angiogenesis, QS-13 also decreases cancer progression by inhibiting tumor cell migration and invasion and *in vivo* tumor growth (Lambert et al., 2018), It would be of interest to propose new therapeutic strategies based for example on QS-13 grafting on the surface of nanoparticles loaded with cytotoxic agents to a specific targeting and drug delivery to the tumor, allowing a decrease in the drug side effects. The patient will benefit from a targeted delivery of therapeutic agents, as well as the anti-angiogenic and anti-tumor activity of QS-13.

## Data Availability Statement

All datasets presented in this study are included in the article/Supplementary Material.

## Ethics Statement

The animal study was reviewed and approved by the French “Ministère de l’Enseignement Supérieur et de la Recherche” (Ethics Committees Nos. C2EA-56 and C2EA-75) in compliance with the “Directive 2010/63/UE”. Protocol no. 4373_V1 APAFIS (07/09/2016).

## Author Contributions

AV-G, JD, CB, SB, LC, AH, AD-D, CS, and SB-P carried out the experiment. JD, SB, LC, BB, J-BO, LR, JM, and SB-P contributed to the interpretation of the results. SB-P took the lead in writing the manuscript. All authors provided critical feedback and helped to improve the manuscript.

## Conflict of Interest

The authors declare that the research was conducted in the absence of any commercial or financial relationships that could be construed as a potential conflict of interest.
