# Supplementary figures and images for: Angiogenesis Inhibition by a Short 13 Amino Acid Peptide Sequence of Tetrastatin, the α4(IV) NC1 Domain of Collagen IV
Source: Front Cell Dev Biol. 2020 Aug 11;8:775. doi: 10.3389/fcell.2020.00775 (PMC7431705; doi:10.3389/fcell.2020.00775)

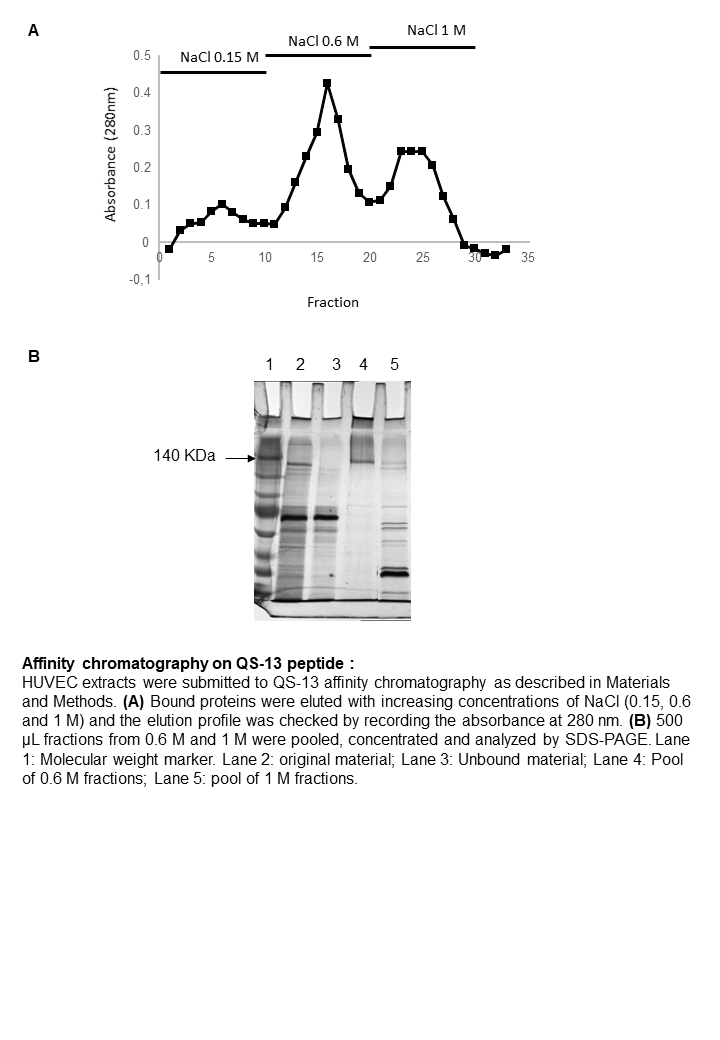

Supplement: Supplementary file 1 [file Image_1.TIF]

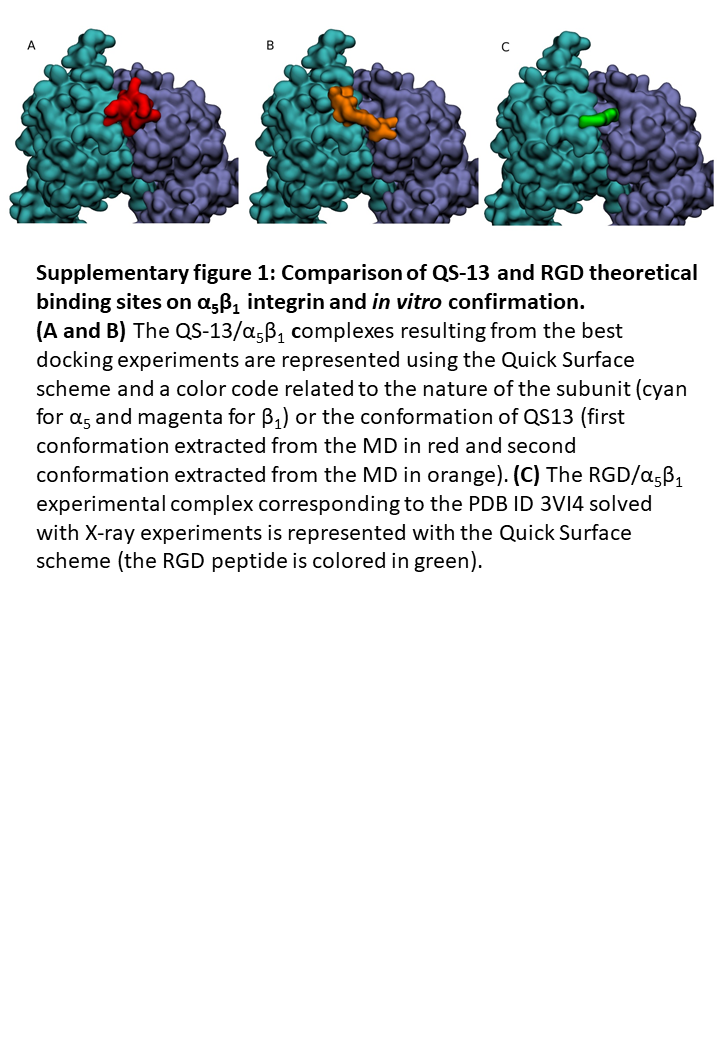

Supplement: Supplementary file 2 [file Image_2.TIF]
